# Whole-genome sequence of *Bacillus subtilis* TR6 strain isolated from tomato (*Solanum lycopersicum* L.) roots

**DOI:** 10.1128/mra.00905-24

**Published:** 2024-10-29

**Authors:** Vladimir K. Chebotar, Maria S. Gancheva, Elena P. Chizhevskaya, Oksana V. Keleinikova, Maria E. Baganova, Alexander N. Zaplatkin, Veronika N. Pishchik

**Affiliations:** 1 All-Russia Research Institute for Agricultural Microbiology, St. Petersburg, Pushkin, Russia; 2 Department of Genetics and Biotechnology, Faculty of Biology, Saint Petersburg State University, St. Petersburg, Russia; The University of Arizona, Tucson, Arizona, USA

**Keywords:** endophyte, *Bacillus subtilis*, tomato, root

## Abstract

Here, we announce the sequence of endophyte *Bacillus subtilis* TR6 strain isolated from tomato roots growing in a greenhouse in St. Petersburg, Russia. With a length of 4,071,622 bp, this strain contained 4,073 potential coding sequences and 10 predicted secondary metabolite biosynthesis gene clusters.

## ANNOUNCEMENT

*Bacillus subtilis* is a rod-shaped, Gram-positive bacterium that has been extensively studied for its potential applications in agriculture, medicine, and industry. In agriculture, it can be used as a biofertilizer or to control plant diseases. Strain TR6 was isolated from the roots of tomato (*Solanum lycopersicum* L.) variety Bella in St. Petersburg, Russia. Tomato roots were disinfected and the TR6 strain was isolated as described previously (1). Genomic DNA was extracted using Wizard Genomic DNA Purification Kit (Promega, USA), and a DNA library was prepared using the KAPA HyperPlus kit (Roche, Switzerland). Whole-genome sequencing was performed using the NextSeq 1000/2000 P2 Reagents V3 (200 cycles), generating paired-end reads (2 × 100 bp). Sequencing was performed at the Lopukhin Center “Genomics, Proteomics, Metabolomics” in Russia.

FastQC v.0.11.9 (2) was used to check the quality of raw reads, followed by assembly using SPAdes v.3.14.1 with the “–careful” option (3). Contigs shorter than 500 bp were removed using a reformat tool from BBMap (https://sourceforge.net/projects/bbmap/). QUAST v.5.1.0 (4) and BUSCO v.5.2.2 (5) were used to measure the assembly metrics. The genome of *B. subtilis* TR6 strain comprised 12 contigs, with a guanine-cytosine content of 43.67% and N50 of 629,767 bp. The reads were mapped to the genome using bwa (6), genome coverage was calculated using the samtools coverage tool (7). The overall genome size was 4,071,622 bp with a coverage of 168×. BUSCO results recovered 99.8% (with 0.2% identified as “Complete and duplicated” and 0.2% as fragmented BUSCOs) of the Bacillales lineage. The taxonomy of TR6 was determined using the FastANI tool (8), which showed 98.7% average nucleotide identity to the *Bacillus subtilis* subsp. *subtilis* ASM904v1. A total of 4,073 and 4,053 potential coding sequences were predicted on the genome assembly by Prokka v.1.14.6 (9) and NCBI Prokaryotic Genome Annotation Pipeline (29 July 2024) (10), respectively. Analysis of secondary metabolite biosynthesis gene clusters was conducted using antiSMASH v.7.1.0 (11) with all extra features turned on and summarized in Table 1. Unless otherwise noted, default parameters were used for all software.

**TABLE 1:** antiSMASH-predicted secondary metabolite biosynthesis gene clusters

Region	Type	From	To	Most similar known cluster		Similarity
Scaffold 1						
Region 1.1	NRP-metallophore, NRPS	81,878	133,655	Bacillibactin	NRP	100%
Region 1.2	CDPS	413,402	434,148	Pulcherriminic acid	Other	100%
Region 1.3	Sactipeptide	655,581	677,192	Subtilosin A	RiPP: thiopeptide	100%
Region 1.4	Other	680,179	721,597	Bacilysin	Other	100%
Scaffold 2						
Region 2.1	Terpene	197,299	218,102			
Region 2.2	TransAT-PKS, PKS-like, T3PKS, NRPS	810,670	925,441	Bacillaene	Polyketide + NRP	100%
Scaffold 3						
Region 3.1	T3PKS	314,100	355,197	1-carbapen-2-em-3-carboxylic acid	Other	16%
Region 3.2	Terpene	403,651	425,549			
Region 3.3	NRPS, betalactone	489,089	566,834	Fengycin	NRP	100%
Scaffold 4						
Region 4.1	NRPS-like	426,471	468,234	K53 capsular polysaccharide	Saccharide	10%
Scaffold 5						
Region 5.1	Lanthipeptide class I	28,955	53,412	Subtilomycin	RiPP: lanthipeptide	100%
Region 5.2	NRPS	180,632	246,020	Surfactin	NRP: lipopeptide	82%

## Data Availability

This project has been deposited at GenBank under accession number PRJNA1141125. The complete genome sequence was deposited under the accession number GCA_041023695.1 and the raw reads in the Sequence Read Archive under the accession number SRR30014060.
